# A Robust Trust Establishment Scheme for Wireless Sensor Networks

**DOI:** 10.3390/s150307040

**Published:** 2015-03-23

**Authors:** Farruh Ishmanov, Sung Won Kim, Seung Yeob Nam

**Affiliations:** 1 Department of Electronics and Communication Engineering, Kwangwoon University, 447-1 Wolgye-dong, Nowon-gu, Seoul 139-701, Korea; E-Mail: farruh.uzb@gmail.com; 2 Department of Information and Communication Engineering, Yeungnam University, 280 Daehak-Ro, Gyeongsan-si 712-749, Gyeongsangbuk-do, Korea; E-Mail: synam@ynu.ac.kr

**Keywords:** trust, attack, misbehavior detection, wireless sensor networks

## Abstract

Security techniques like cryptography and authentication can fail to protect a network once a node is compromised. Hence, trust establishment continuously monitors and evaluates node behavior to detect malicious and compromised nodes. However, just like other security schemes, trust establishment is also vulnerable to attack. Moreover, malicious nodes might misbehave intelligently to trick trust establishment schemes. Unfortunately, attack-resistance and robustness issues with trust establishment schemes have not received much attention from the research community. Considering the vulnerability of trust establishment to different attacks and the unique features of sensor nodes in wireless sensor networks, we propose a lightweight and robust trust establishment scheme. The proposed trust scheme is lightweight thanks to a simple trust estimation method. The comprehensiveness and flexibility of the proposed trust estimation scheme make it robust against different types of attack and misbehavior. Performance evaluation under different types of misbehavior and on-off attacks shows that the detection rate of the proposed trust mechanism is higher and more stable compared to other trust mechanisms.

## 1. Introduction

Trust establishment is one of the more recent research trends in many fields, such as web-based services, e-commerce, peer-to-peer networks, and wireless networks. In wireless sensor networks (WSNs), different trust establishment (TE) methods, technologies and mechanisms, such as fuzzy logic [1,2], bio-inspired [3,4,5], and deterministic- and probabilistic-based approaches [6,7,8,9,10,11,12,13], have recently been proposed. In general, TE can be used in WSNs for two purposes: cooperation improvement and security enhancement [7,10,11,12]. Cooperation among sensor nodes in WSNs is vital to maintaining the operation of the network [14,15]. This shows the importance of maintaining collaboration among sensor nodes. Collaboration can be successful when all nodes operate in a reliable manner [6,7]. TE maintains successful collaboration by detecting reliable and unreliable nodes and assessing them based on their actions/performance.

Moreover, because WSNs are usually deployed in remote and unattended areas, and nodes are usually not tamper-resistant, they can be physically captured and are easily compromised. Once a node is compromised, security techniques like cryptography and authentication fail to protect the network. Thus, TE can continuously monitor and evaluate node behavior and detect such compromised nodes. The relationships of other features of WSNs to attack and misbehavior are summarized in Table 1.

**Table 1:** Relation between attacks and features of wireless sensor networks.

Features of WSNs	Relation to Attacks and Misbehavior
Deployment environment (open, unattended environment)	Nodes can be physically captured and easily compromised. Compromised nodes can launch sophisticated attacks against trust establishment.
Low-cost nodes	This causes nodes to often get stuck malfunctioning due to software and hardware problems, which requires trust establishment to have features to detect such misbehavior.
Self-organized	Groups of malicious nodes can organize collaborative attacks against trust establishment.
Diverse applications	This implies that attack and misbehavior type, intensity, strategy, frequency, *etc.*, can vary according to the application.

Although a lot of research has been proposed, the robustness of TE has not received enough attention from researchers. Just like security schemes, TE itself is vulnerable to attack. Because one objective of a malicious node is not to be detected while attacking, the node can adopt different strategies for an on-off attack. Although some techniques have been proposed to alleviate an on-off attack [7,11,13], to the best of our knowledge, they cannot efficiently tackle different strategies for an on-off attack. Moreover, malicious nodes can persistently and intentionally maintain fewer bad behaviors compared to number of good behaviors, so they are not detected while slowly damaging the network. This issue is also not addressed in previous research.

Considering the above-mentioned problems and resource-constrained sensor nodes, we propose a robust yet lightweight TE scheme. Specifically, the robustness and light weight of the proposed TE scheme arise from the following:

(1) Unlike traditional TE schemes, ours introduces a new component in trust estimation, which we call misbehavior frequency. Misbehavior frequency can tackle different strategies for an on-off attack. In addition, it helps the network detect and uncover persistent malicious nodes. Moreover, depending on the performance of the node, trust estimation adapts different equations to estimate trust in order to mitigate the effects of on-off attacks. Another important feature of the proposed scheme is that it can differentiate between legitimate and malicious nodes. Hence, it can avoid false accusations against a legitimate node while maintaining efficient detection of malicious nodes.
(2) Although we introduce a new component (frequency of misbehavior), computational overhead in terms of the number of operations is lower compared to some previous mechanisms (see Appendix). Thanks to the misbehavior-frequency component, the proposed trust mechanism is robust.

Comprehensive performance evaluation results show that the proposed scheme can more efficiently detect different misbehavior and on-off attacks more efficiently, compared to other methods. Specifically, under different strategies for an on-off attack, the proposed method demonstrates a higher and more balanced detection rate compared to previously proposed schemes. In addition, evaluations in terms of false-positive and false-negative alarm rates demonstrate that the proposed TE scheme can differentiate between a legitimate and a malicious node. Although proposed scheme is more sensitive to false-positive alarm compare to other schemes as evaluation results show, dynamic optimal trust threshold can be set according to the network scenario and performance of trust mechanism, which will not only avoid false-positive alarms but also optimize the performance of trust mechanism. Instead of performing evaluations under optimal trust threshold we use intuitive trust threshold value used by previous research works in order to be fair with other trust mechanisms. Hence, performance evaluations demonstrate that proposed scheme is more sensitive to false-positive alarm compare to other schemes. The remainder of this paper is organized as follows: Related work is discussed in Section 2. Section 3 presents the proposed trust establishment method. Section 4 presents performance evaluation, and Section 5 concludes the paper.

## 2. Related Work

As proposed by Ishmanov *et al.* [16], trust establishment schemes in WSNs can be divided into the following groups based on trust estimation method:

- Probabilistic
- Fuzzy logic
- Weighting
- Miscellaneous

Below are some representative examples of those TE schemes. One of the earliest state-of-the-art TE methods was proposed by Shaikh *et al.* [10], called the group-based trust management scheme (GTMS) for clustered wireless sensor networks. The scheme works on three levels: the node level, the cluster head level, and the base station level.

**Figure 1:**
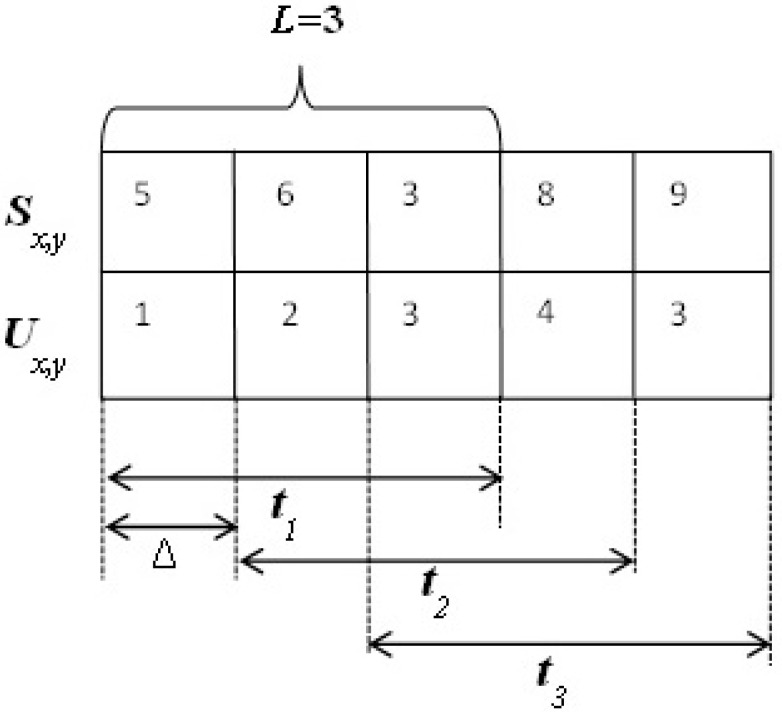
Example of the time-window mechanism.

At the node level, nodes estimate a trust value for other nodes using a timing-window mechanism. The main objective of the timing window is to record and forget previous records. After each Δ period, node *x* estimates the trust value of node *y* based on the information recorded in time window *t_k_*. As the example in Figure 1 shows, after each Δ period, the time window slides to the right, recording recent information and forgetting information recorded earlier. The time window in Figure 1 consists of three time units (*L* = 3), and *S_x,y_* and *U_x,y_* are good and bad behavior, respectively, of node *y* observed by node *x* within time window *t_k_*.

Using the information in the time window, the trust value of node *y* per node *x* is estimated as follows [10]:

(1) $$T_{x,y}=\left[100\times \left(\frac{{\left(S_{x,y}\right)}^{2}}{\left(S_{x,y}+U_{x,y})(S_{x,y}+1\right)}\right)\right]$$

where [·] is the nearest integer function, *S_x,y_* is the total number of successful interactions by node *x* with node *y* during time *t_k_*, and *U_x,y_* is the total number of unsuccessful interactions by node *x* with node *y* during time *t_k_*. After estimation of the trust value, a node will quantize trust into three states in the proposed mechanism: trusted, uncertain, and untrusted.

Advantages of this scheme are that it is lightweight and energy-aware, both of which meet the requirements of WSNs. Furthermore, the authors proved that GTMS is resilient against cheating, bad behavior, and group attacks, under the assumption that the number of unsuccessful interactions is equal to, or more than, the number of successful interactions. However, this may not always be true, because an attacking node usually attempts to avoid detection as much as possible. Moreover, the time window is not resilient enough to counter on-off attacks.

Maturity-based trust management for mobile *ad hoc* networks was proposed by Velloso *et al.* [12]. The relationship maturity concept was introduced to improve the quality of a trust evaluation in the presence of mobility. According to the concept, recommendations by long-term neighbors are given more weight than recommendations by short-term neighbors. The trust level of node *y* is estimated by node *x* by combining observation-based trust with recommendations as follows [11]:

(2) $$T_{x}(b)=(1-\text{α})\times Q_{x}(b)+\text{α}\times R_{x}(b)$$

where *Q_x_*(𝑏) is an observation-based trust value from node *x* about node *y*, and 𝑅*_x_*(𝑏) represents the aggregate value of the recommendations from all neighbors. The variable α provides a relevant weight to each factor. *Q_x_*(𝑏) is defined as follows [11]:

(3) $$Q_{x}(b)=\text{β}\times E_{x}(b)+(1-\text{β})\times T_{x}(b)$$

where 𝐸*_x_* and 𝑇*_x_* are current and previously obtained trust values, respectively. The variable β provides a necessary weight to each trust value.

The merit of this proposed method is that it can maintain trust establishment in a mobile environment. However, the proposed scheme is not immune to on-off attacks because it has no inbuilt technique against an on-off attack.

One recent trust establishment scheme, an attack-resistant and lightweight trust management approach for medical sensor networks called ReTrust, was proposed [11]. Node *x* calculates a trust value for node *y* using a time window as follows [11]:

(4) $$T_{x,y}=\left[\text{α}\times \left(\frac{\sum_{j=1}^{m}{\text{β}}_{j}\times (1-p_{j})\times p_{j}}{\sum_{j=1}^{m}{\text{β}}_{j}\times (1-p_{j})}\right)\right]$$

where α scales the range of the trust value, and *m* is the number of units in a time window. β*_j_* is an aging-factor parameter. β*_j_* is defined as β*_j_ =* φ*^L−j^*, where *0 <* φ *< 1*. This means that the forget factor is different for each time unit *j*. *p_j_* is a successful interaction rate, which is calculated as follows [11]:

(5) $$p_{j}=\frac{S_{j}+1}{S_{j}+U_{j}+2}$$

where *S_j_* and *U_j_* are the number of successful and unsuccessful interactions, respectively, during the *j*th unit of the time window.

Using the time-window mechanism along with the proposed comprehensive aging mechanism makes the trust estimation robust against an on-off attack. However, like traditional trust estimation methods, ReTrust also does not take into account persistency of misbehavior.

Banković *et al.* [17] proposed an intrusion detection system based on a reputation system. One idea behind the proposal is to use a self-organizing map (SOM) algorithm to produce a reputation based on the performance of the monitored node. Specifically, an agent residing on each node monitors neighboring nodes for data consistency and uses a SOM algorithm to analyze and find any abnormality in sensor values. Hence, if an agent detects nodes that report inconsistent data with neighboring nodes, it will assign a lower reputation. The following equation is used to update the reputation of a node [17]:

*new reputation* [*node*] = *last_som_reputation* [*node*] + curRep + log (0.99 × curRep) (6)

where *last_som_reputation* [*node*] and *curRep* are the last and the current reputation values of the node, respectively. If the produced reputation value is greater than 1, it will be truncated to 1, and if it is lower than 0, it will be truncated to 0. The function *x* + log (0.99*x*) regulates the increase and decrease of the reputation value accordingly. The last and current reputation values are derived using the SOM algorithm. Details of the derivation are given elsewhere [17]. If a node has a elsewhere low reputation value, then messages sent by this node are discarded so that it is isolated. The merit of this scheme is that the produced reputation values are accurate due to the SOM algorithm. Moreover, isolation of malicious nodes is another meritorious aspect of the scheme. The application-specific aspects of the scheme, such as detection of abnormal sensor data, might limit application range. For example, intentional packet drops by malicious nodes cannot be tackled by the proposed reputation system.

Another interesting trust and reputation model was proposed by Marzi and Li, which is enhancement to a bio-inspired trust and reputation model for wireless sensor networks [18]. The bio-inspired algorithm of an ant colony system (ACS) is used to establish trust and reputation among nodes. Specifically, when the algorithm is launched, a set of artificial “*ants*” are deployed over the network. These “*ants*” move over the network from source to destination to find the most trustworthy path. Once the “*ants*” have found a path leading to a destination node, a score has to be given to each of those paths and reported to the source node. During their travels, the “*ants*” modify pheromone values (trust values) on nodes, depending on the quality of the paths between nodes. Moreover, upon arrival at each node, the “*ant*” decides on the action to take, depending on the ACS algorithm and situation. In this way, the “*ants*” help source nodes to find the most trustworthy route to a destination node. Convergence time to find a reliable path from source to destination might be high while the “*ants*” travel the network until they find the optimal route. Hence, the proposed scheme might not be suitable for delay-sensitive applications. Moreover, constrained resources of WSNs, such as energy, bandwidth, and computation for the ACS algorithm to be run on sensors, should be considered.

## 3. Proposed Trust Estimation Method

### 3.1. Assumptions and Considerations

We assume that nodes can observe the activities of other nodes within communication range. For example, a node can overhear its neighbors’ transmissions, and in this way, can detect whether the node is forwarding or dropping packets. Note that we do not consider trust value exchange among nodes. Hence, nodes estimate trust values based on monitoring only. Moreover, we assume that all nodes are static. A malicious node misbehaves in a smart way; that is, it tries to maintain a high trust value while misbehaving. We also assume that all nodes have unique identities, and authentication methods are used to defend against using a fake ID. In order to propose generic trust establishment, we avoid application-specific features in our trust estimation, following previous research works [4,10,11,12,13]. However, in order to adapt easily to some specific applications, we leave adaptation room in the trust estimation equation. Specifically, θ and β parameters are to serve the application and network needs*.* Hence, the proposed trust scheme can be applied easily, provided there is frequent or periodic interaction among nodes. Moreover, we assume that a technique/method exists to distinguish between good and bad behavior. For example, if trust establishment is applied in data aggregation, data outlier techniques can be used to distinguish between false/incorrect and true/correct data.

### 3.2. Robust Trust Estimation Method

**Figure 2:**
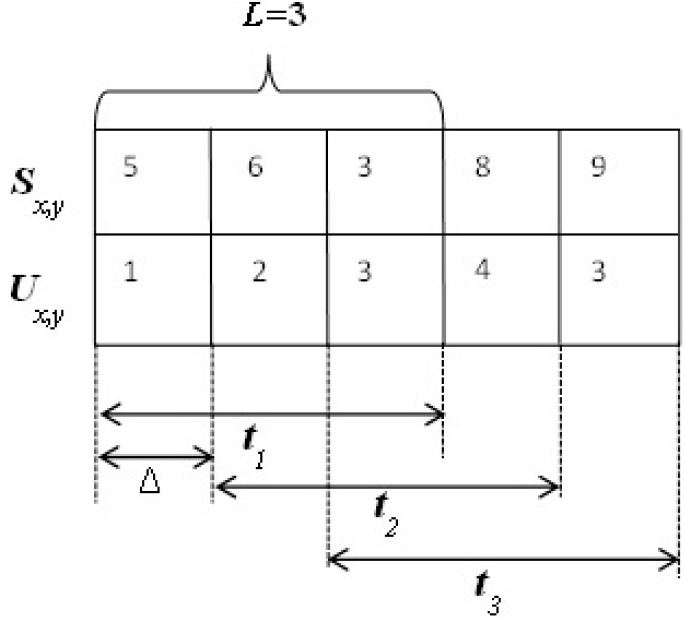
Application of the time-window mechanism in managing and recording observations.

Traditionally, trust is estimated based on the observed weight of misbehavior. In order to record and manage observations, we use a time-window mechanism. Figure 2 illustrates an example of time-window usage. According to Figure 2, node *x* records observations about node *y*, in which *S_x_*_,*y*_ and *U_x_*_,*y*_ are the numbers of good and bad behaviors, respectively, of node *y* as observed by node *x*. Moreover, the time window consists of three time units, *L* = 3. After each Δ time period, the time window slides to the right, adding a new time unit and deleting the very first time unit, as Figure 2 demonstrates.

Based on the rate of misbehavior in each time unit, node *x* estimates the weight of misbehavior as follows:

(7) $$w_{t_{k}}^{m}=\text{max}(\alpha_{1}r_{1},\alpha_{2}r_{2},....,\alpha_{j}r_{j},\;\alpha_{L}r_{L})$$

where $w_{t_{k}}^{m}$ is the weight of misbehavior for time *t_k_*. The variable α, which is in the range (0, 1), represents the forget factor. It assigns less weight to the older measured misbehavior so that recent performance is given more importance. Hence, α_1_ < α_2_ < … < α*_L_*. An exponential decrease function is used to forget old observations. More specifically, α*_j_ =* φ*^L−j^*, where 0 < φ < 1. Here, the max function helps to remember a greater rate of misbehavior for a long time. The objective of such a method is to defend against an on-off attack because, in an on-off attack, a malicious node changes its action from bad to good, and vice versa, so it is not detected. Hence, when a malicious node intends to misbehave in one time unit and show good behavior in another time unit, the proposed method helps to alleviate the effect of the on-off attack. *r_j_* is the rate of misbehavior in time unit *j*. The rate of misbehavior for time unit *j* is estimated as follows:

(8) $$r_{j}=\left\{ \begin{array}{l} 0\;if\;\frac{U_{j}}{U_{j}+S_{j}}\leq \text{θ}\\ \frac{U_{j}}{U_{j}+S_{j}}\;otherwise \end{array}\right.$$

where *θ* is an application-specific or network scenario–specific parameter. The purpose of this parameter is to avoid the effect on trust of the network’s condition, or to accommodate the application’s needs. For example, if trust establishment is applied in routing, and trust is estimated based on the number of forwarded and dropped packets, then dropped packets due to channel conditions or collisions should not affect the trust value. *S_j_* and *U_j_* are the numbers of good and bad behaviors, respectively, in time unit *j*.

Then, trust is estimated using a measured weight of misbehavior as follows:

(9) $$T_{t_{k}}=1-w_{t_{k}}^{m}$$

Depending on the weight of the misbehavior, the trust value varies from zero to one, just as the weight of misbehavior varies from zero to one. The proposed trust estimation method is simple and lightweight. Being lightweight is an important requirement for algorithms in WSNs, because sensor nodes are limited in terms of computational and energy capability. Although this method is lightweight and robust, it does not consider the frequency of misbehavior in trust estimation. In some cases, using only the weight of the misbehavior does not allow correct evaluation of the node. For example, a node might be stuck malfunctioning, and persistently misbehaves. In this case, if the weight of misbehavior is low, traditional trust estimation methods always assign a high trust value even though the node misbehaves persistently for a long time. Moreover, a malicious node might act intelligently, such as launching insignificant but persistent attacks, so it is not covered by the trust estimation method. In addition, traditional trust mechanisms cannot fully cope with an on-off attack [7]. Hence, we enhance our trust estimation method by incorporating a frequency-of-misbehavior component.

Frequency of misbehavior shows how frequently a node misbehaves during a certain time interval. We use a time-window mechanism to estimate the frequency of misbehavior. It is measured based on the number of on and off periods during the *t_k_* period*.* Because time window *t_k_* has several time units, each time unit *j* is defined as either an on period or an off period based on the rate of misbehavior in time unit *j* as follows:

(10) $$j=\left\{ \begin{array}{l} \text{on-perod}\;if\;r_{j}>\text{θ}\\ \text{off-period}\;otherwise \end{array}\right.$$

So, if the rate of misbehavior is greater than a certain threshold, θ, then time unit *j* is defined as an on period. Otherwise, it is considered an off period.

**Figure 3:**
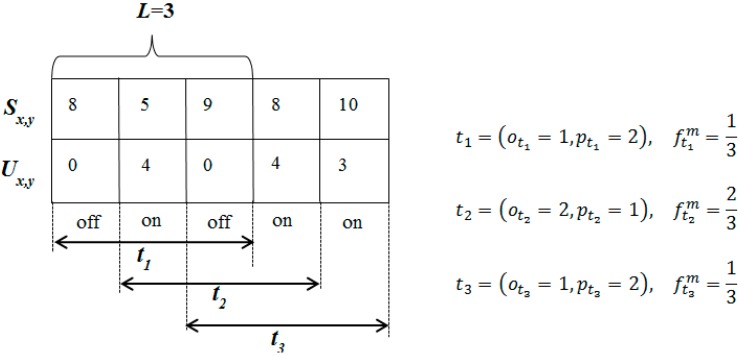
Misbehavior frequency estimation using a time-window mechanism.

After defining all the time units as either an on or off period within the *t_k_* period, based on the number of on and off periods, the frequency of misbehavior is measured as follows:

(11) $$f_{t_{k}}^{m}=\frac{o_{t_{k}}}{o_{t_{k}}+p_{t_{k}}}$$

where $o_{t_{k}}$ and $p_{t_{k}}$ are the number of on and off periods during *t_k_*. In order to update the frequency of misbehavior after each Δ time period, the time window slides to the right, forgetting information in the first time unit and adding information in the last time unit. A sample scenario of time-window usage to estimate frequency of misbehavior is illustrated in Figure 3.

According to Figure 3, node *x* records observations and estimates the frequency of misbehavior for node *y*. For the sake of simplicity, a threshold value is set to θ = 0, and the number of time units in the time window (*L*) is 3. As seen in Figure 3, each time unit is defined as either an on or off period depending on the misbehavior rate. For example, in the first time period, there is no misbehavior (that is, the misbehavior rate is zero), so it is defined as an off period. Based on the number of on and off periods in each time window, the frequency of misbehavior is estimated for *t_1_*, *t_2_*, and *t_3_*. Then, the node’s status is determined according to $f_{t_{k}}^{m}$ as follows for time window *t_k_*:

(12) $$S\left(f_{t_{k}}^{m}\right)=\{\begin{array}{l} 1\;\text{persistent malicious node }\\ (0;\text{θ})\;\text{legitimate node}\\ (0;1)\;\text{malicious or on-off attacking node } \end{array}\}$$

A node is considered a persistent malicious node if all time units are found to be an on period; that is, $f_{t_{k}}^{m}=1$. On the other hand, if all time units are off periods, $f_{t_{k}}^{m}\in (0;\text{θ})$ then a node is considered legitimate, or a good node. If the value of $f_{t_{k}}^{m}$ is between (1;θ) then the node is considered malicious, or an on-off attack node.

Measured frequency and weight of misbehavior are combined to obtain trust value $T_{t_{k}}$ for time window *t_k_* as follows:

(13) $$T_{t_{k}}=\left\{ \begin{array}{l} 1-w_{t_{k}}^{m}\;if\;w_{t_{k}}^{m}>f_{t_{k}}^{m}\\ \beta \times \left(1-f_{t_{k}}^{m}\right)+\left(1-\beta \right)\times \left(1-w_{t_{k}}^{m}\right)\;otherwise \end{array}\right.$$

where β is the weight given to the frequency and weight of misbehavior, which varies in the range [0.5;1]. Depending on the application or performance requirement, different β values are assigned to each factor. For example, if frequency of misbehavior is more important than weight of misbehavior (instantaneous misbehavior) for some applications, then more weight is given to frequency of misbehavior. Hence, our scheme provides room for adaptability. Moreover, trust estimation includes two terms. According to the situation, trust estimation adopts a different equation to estimate trust. The goal of such a design is to avoid tricks of a malicious node. For example, a malicious node might intend to attack fewer times but more heavily. In this case, since the value of the frequency of misbehavior will be low, the malicious node will obtain a higher trust value if that trust value is obtained using the second term. Hence, such a design provides robustness to the trust estimation. After each Δ period, each node estimates three components: frequency and weight of misbehavior, and trust. Moreover, after each Δ period, the number of on and off periods is updated using the sliding time window.

## 4. Performance Evaluation

In this section, we present the results of our evaluation and comparisons of the proposed trust scheme against earlier proposed schemes. We evaluated it in terms of detection of different persistent malicious behaviors and different strategies for on-off attacks. Comparisons were done with GTMS [10] and ReTrust [11].

Values of the trust scheme parameters, such as trust threshold, forget factor, and number of time units in the time window, were chosen based on heuristic and previously defined values in the literature. For instance, trust threshold was chosen at about half of the maximum trust value used in various other studies [10,19,20,21,22,23,24]. Those papers defined the trust threshold at between 0.4 and 0.8, where trust values range between 0 and 1. For Yu *et al.* [21], the most intuitive trust threshold is 0.5. In another scenario from Bao *et al.* [24], the optimal trust threshold is 0.6. The value of the forget factor is often selected heuristically and depends on many factors, such as application, preference, situations, *etc.* [21]. Since the goal of the forget factor is mainly to mitigate the effect of an on-off attack, different authors proposed using different values and different equations to derive the value of the forget factor [7,10,20,22]. Following the guidelines and suggestions of Sun *et al.* [7], we choose 0.7 as the forget factor.

### 4.1. Node Behavior Modeling

In order to evaluate trust establishment under node misbehavior, we first need to define behavior of a benevolent node and misbehavior in a node. Hence, in this section, we define general and basic notions about benevolent and malicious nodes, and we model node behavior. Note that in this modeling, we do not differentiate between malfunctioning and malicious nodes. We call them malicious nodes, in general.

Ideally, a benevolent node always behaves well, except that sometimes it might misbehave temporarily due to different factors. For example, sometimes a forwarding node might temporarily drop packets due to channel conditions. Another example is where a node always reports correct sensor data but might sometimes also reports incorrect sensor data due to a computation error or a sensing error. Note that behavior of a benevolent node can be similar to behavior of an on-off attacking node, in which the malicious node changes its behavior from bad to good, and vice versa. However, in an on-off attack, a bad behavior pattern is predetermined and comprehensive. On the other hand, misbehavior of a benevolent node is random and depends on different factors, as mentioned above. Hence, an important point about a benevolent node’s behavior is that the misbehavior is random and temporary.

A malicious node demonstrates persistently bad behavior, and the rate of misbehavior can be either significant or insignificant. This kind of assumption is important in WSNs, because research studies show that a sensor node often becomes stuck malfunctioning [8]. Moreover, when misbehavior is significant, its detection is easy and obvious. Hence, a malicious node might intentionally demonstrate persistent and insignificant misbehavior so it is not detected while attacking. Hence, an important point about the behavior of a malicious node is that it persistently and intentionally demonstrates misbehavior.

In order to model node behavior according to the above definitions and to be more natural and generic, we use binomial distribution. Since binomial distribution has the following properties, it fully satisfies our modeling requirements:

- It consists of a sequence of *n* identical trials.
- Two outcomes, success or failure, are possible on each trial.
- The probability of success on any trial, denoted *p*, does not change from trial to trial.
- The trials are independent.

Trials in binomial distribution can take the total number of behaviors/interactions/actions of the node during the monitored period into consideration. The probability of each behavior/interaction/action being malicious/misbehavior is 1 − *p* = *q.* On the other hand, the probability of each behavior/interaction/action being legitimate/good behavior is *p.* The independence of each behavior/interaction/action and the independence of the outcome of each behavior/interaction/action from previous outcomes make modeling more natural and generic.

### 4.2. Misbehavior Detection

To create a simulation according to the above defined behavior of a malicious node for each time unit of the time window, 10 behaviors were generated. When *p* ≥ 0.9, it demonstrates the behavior of an ideal and benevolent node. In other cases, we assume that it demonstrates the behavior of a malicious node. Hence, selected values for *p* are *p* ≥ 0.6 and *p* ≥ 0.5. For each behavior, a random number is generated between 0 and 1. If the generated number is equal to or smaller than 0.6, then the behavior is considered bad; otherwise it is counted as good. Hence, the numbers of good and bad behavior are determined in this way for each time unit, and trust is estimated using the parameters in Table 2.

**Table 2:** Misbehavior detection simulation parameters.

Parameter	Value
Number of time units in time window	*L* = 3
Number of behaviors in each time unit	10
Trust and misbehavior frequency and weight estimation period	Δ
Trust threshold	*S* = 0.6
Simulation time	100Δ
Beta value	*Β* = 0.7
Forget factor	*α* = 0.7 (for all trust schemes)
Threshold for rate of misbehavior	θ *=* 0.1
Probability of good behavior	*p* *≥* 0.6, *p* *≥* 0.5

**Figure 4:**
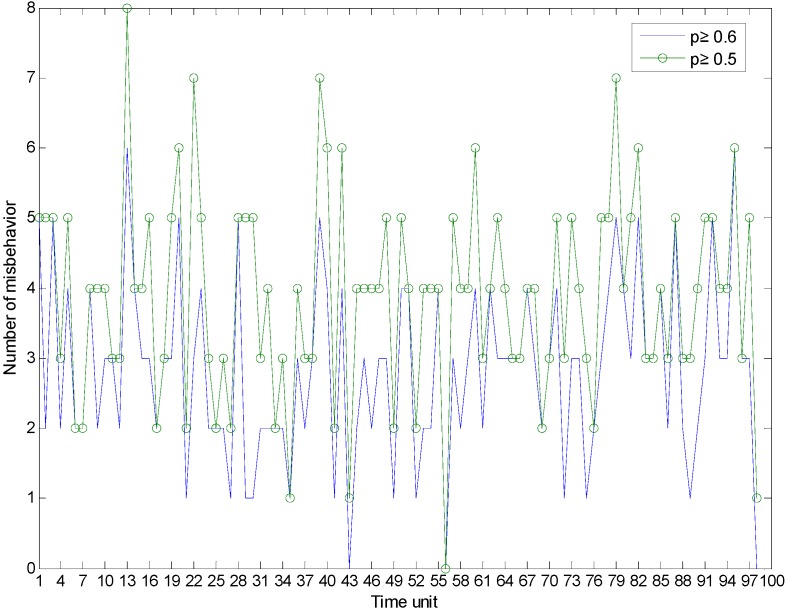
Generated number of misbehaviors.

**Figure 5:**
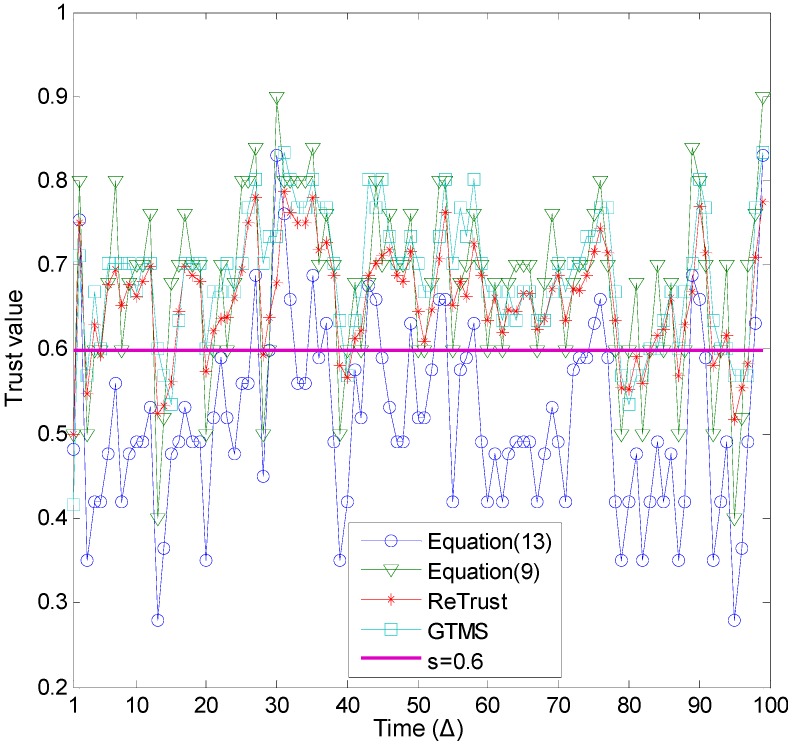
Misbehavior detection (*p* ≥ 0.6).

Figure 4 demonstrates the generated number of misbehavior for each time unit, with different probabilities for good behavior. On average, the number of misbehavior is two to five out of 10 behaviors. Figure 5 illustrates the trust estimation for each time unit of the time window when *p* ≥ 0.6. In order to show the benefit of the introduced misbehavior-frequency component, trust is estimated with and without the misbehavior-frequency component. In Equation (9), we consider only the weight of the misbehavior. The performance of the proposed trust mechanism with Equation (9) is quite similar to other compared trust mechanisms, as Figure 5 demonstrates. However, the proposed trust mechanism with Equation (13) outperforms other trust mechanisms.

**Figure 6:**
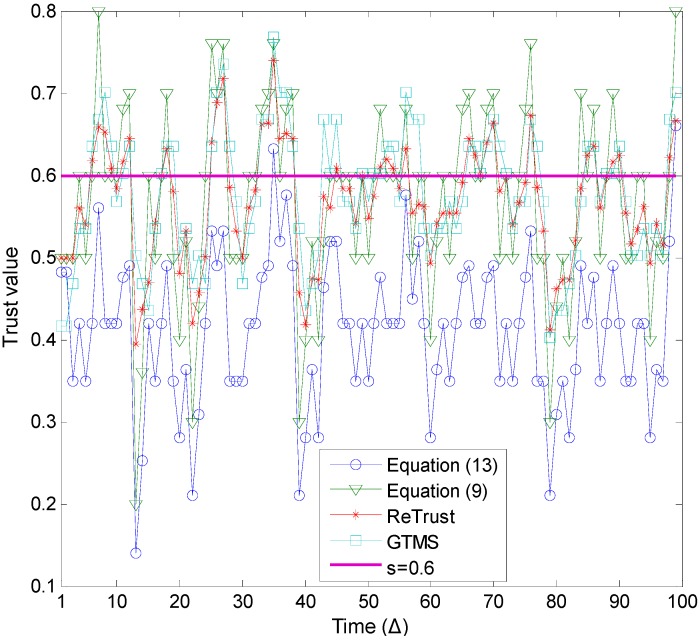
Misbehavior detection (*p* ≥ 0.5).

Figure 6 illustrates the trust estimation for each time unit of the time window when *p* ≥ 0.5. A general observation from this figure is that, as the number of misbehaviors increases in the evaluations of Figure 6, the estimated trust values of all trust mechanisms are lower compared to the results in Figure 5. As a consequence, trust mechanisms can detect more misbehavior in this case. In addition, the proposed trust mechanism with Equation (13) outperforms all other trust mechanisms.

### 4.3. False-Positive and False-Negative Alarm Rates

A false-positive alarm rate indicates the total number of times a legitimate node is found to be a malicious node, divided by the total number of evaluations. In other words, it indicates how often trust establishment accuses legitimate nodes of being malicious. On the other hand, the false-negative alarm rate is defined as the total number of times a malicious node is deemed legitimate, divided by the total number of evaluations.

In order to evaluate and compare the proposed mechanism in terms of false-positive alarm rate, we used binomial modeling of an ideal legitimate node, in which *p* is equal to or greater than 0.9. Moreover, since such a value of *p* is for ideal cases, *p* might have smaller values depending on the conditions. For example, if we assume that *p* is packet-forwarding probability, then packet drops might increase due to collision or channel conditions. In such a situation, *p* of a legitimate node can be smaller than 0.9. Such conditions should not have an impact on the legitimacy of the node. Thus, we evaluate and compare situations when *p* is equal to or greater than 0.8 and 0.7. Parameters in Table 3 are used to simulate them.

**Table 3:** False-positive and false-negative alarm rate evaluation parameters.

Parameter	Value
Number of time units in time window	*L* = 3
Number of behaviors in each time unit	10
Trust and misbehavior frequency and weight estimation period	Δ
Trust threshold	s = 0.6
Simulation time	100Δ
Beta value	β = 0.7
Forget factor	*α* = 0.7 (for all trust schemes)
Threshold for rate of misbehavior	θ *=* 0.1, θ *=* 0.2, θ *=* 0.3
Probability of good behavior	*p* *≥* 0.9, *p* *≥* 0.8, *p* *≥* 0.7, *p* *≥* 0.6, *p* *≥* 0.5, *p* *≥* 0.4

**Figure 7:**
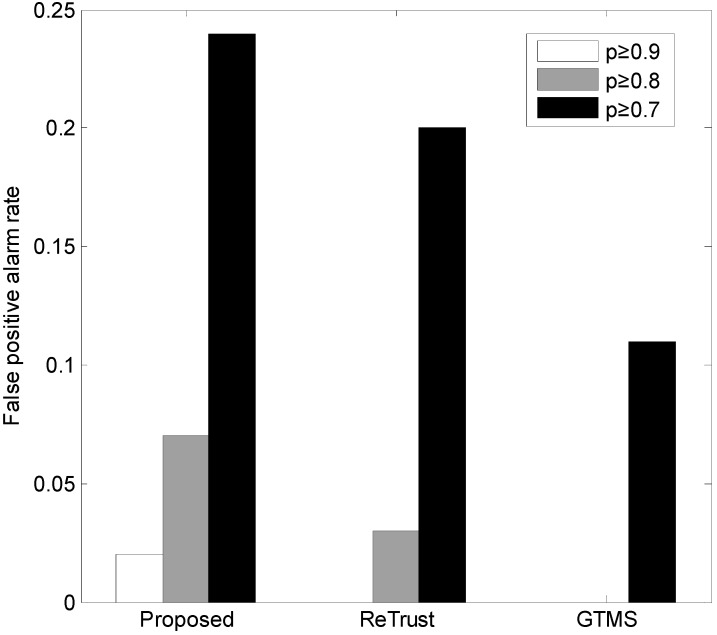
False-positive alarm rate.

As Figure 7 shows, the proposed trust establishment has a slight false-positive alarm in ideal cases. Specifically, out of 100 time evaluations, there are two false-positive alarms. In general, the proposed scheme is more sensitive to false-positive alarms, compared to the other schemes. An important observation is that none of the trust schemes is enabled with a technique against false-positive alarms, as Figure 7 shows.

**Figure 8:**
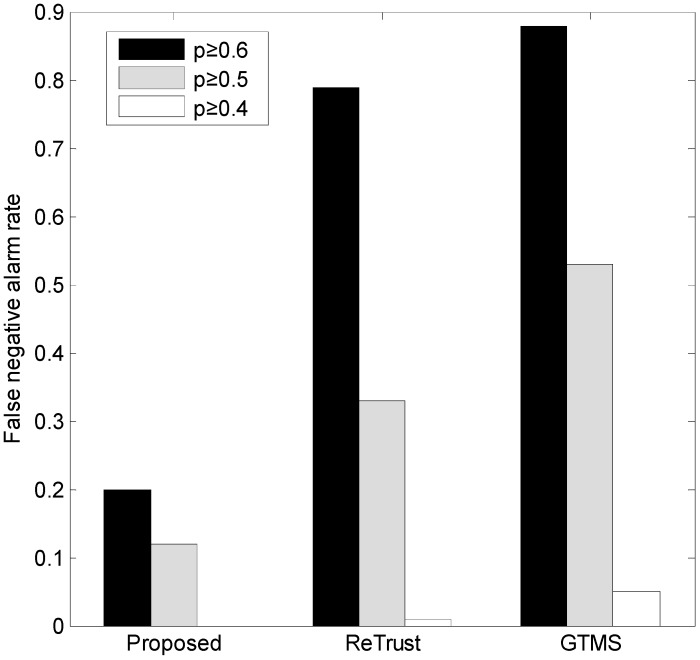
False-negative alarm rate.

Figure 8 illustrates the results of our evaluation in terms of false-negative alarm rate with different values of *p.* A general observation is that when *p* decreases, the false-negative alarm rate also decreases. Because the number of bad behaviors increases in this case, detection of misbehavior becomes obvious. The proposed scheme shows a very low false-negative alarm rate compared to the others.

### 4.4. On-Off Attack Detection

In this section, we evaluate and compare our trust scheme under on-off attacks. Parameters in Table 4 are used to simulate the behavior of an on-off attack node.

**Table 4:** Parameters to simulate an on-off attack.

Parameter	Value		
Probability of an on period	0.6, 0.4, and 0.2		
Probability of an off period	0.4, 0.6, and 0.8		
Number of good behavior	On period:	Randomly generated between:
	0.6	8 to 10
	0.4	8 to 10
	0.2	8 to 10
	Off period:	Randomly generated between:
	0.4	8 to 10
	0.6	8 to 10
	0.8	8 to 10
Number of bad behavior	On period	Randomly generated between:
	0.6	4 to 6
	0.4	6 to 9
	0.2	12 to 18
	Off period	In all cases, zero
Number of time units	*L* = 3 (for other trust schemes); *L* = 10 (for the proposed trust scheme)	
Trust and misbehavior frequency and weight estimation period	Δ	
Trust threshold	*s* = 0.6	
Experiment time	100Δ	
Weight parameter	β = 0.7	
Forget factor	α = 0.7	
Threshold for rate of misbehavior	θ = 0	

To make the simulation more realistic and fair, we used three different types of on-off attack. In the first type, a malicious node intends to attack more frequently, but decreases the number of bad behavior. In other words, the frequency of the misbehavior increases, but the weight of the misbehavior decreases. In this type of attack, the probability of an on period is set to 0.6, and the numbers of good and bad behavior are generated between 8 and 10 and 4 and 6, respectively, during an on period. Moreover, on and off periods are randomly distributed over time. In the second type of on-off attack, a malicious node intends to attack fewer times, compared to the first type, but it increases the number of bad behavior during the on periods. So, in this strategy, the probability of an on period is set to 0.4, and the numbers of good and bad behavior are generated between 8 and 10 and 6 and 9, respectively, during an on period. Finally, in the third type of on-off attack, a malicious node intends to attack the least, compared to the previous types, but the number of bad behavior is the highest for each attack time, compared to the previous types. Hence, the probability of an on period is set to 0.2, and the numbers of good and bad behavior are generated between 8 and 10 and 12 and 18, respectively, during an on period. In all three strategies, the number of good behavior is generated randomly at between 8 and 10, and the number of bad behavior is always 0 during an off period.

**Figure 9:**
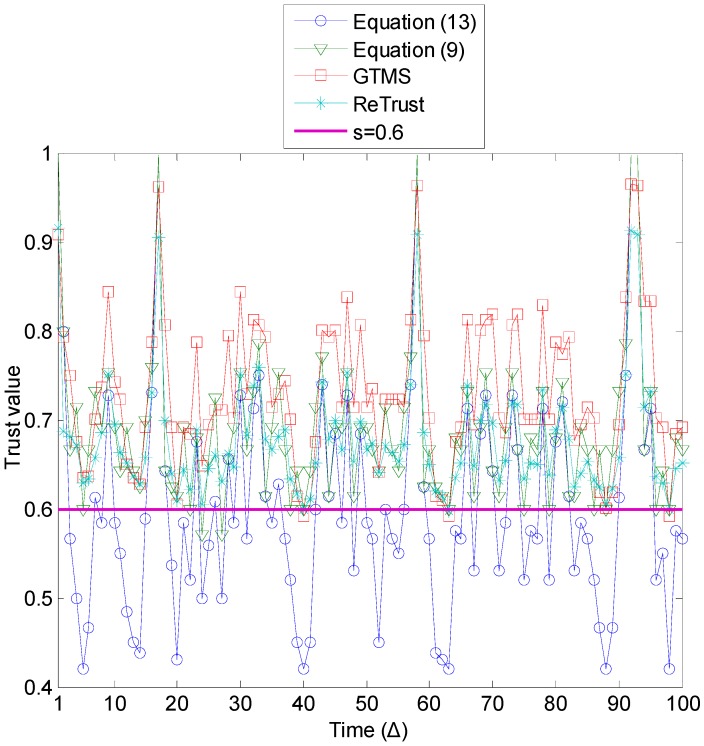
On-off attack detection (probability of an on period is 0.6).

**Figure 10:**
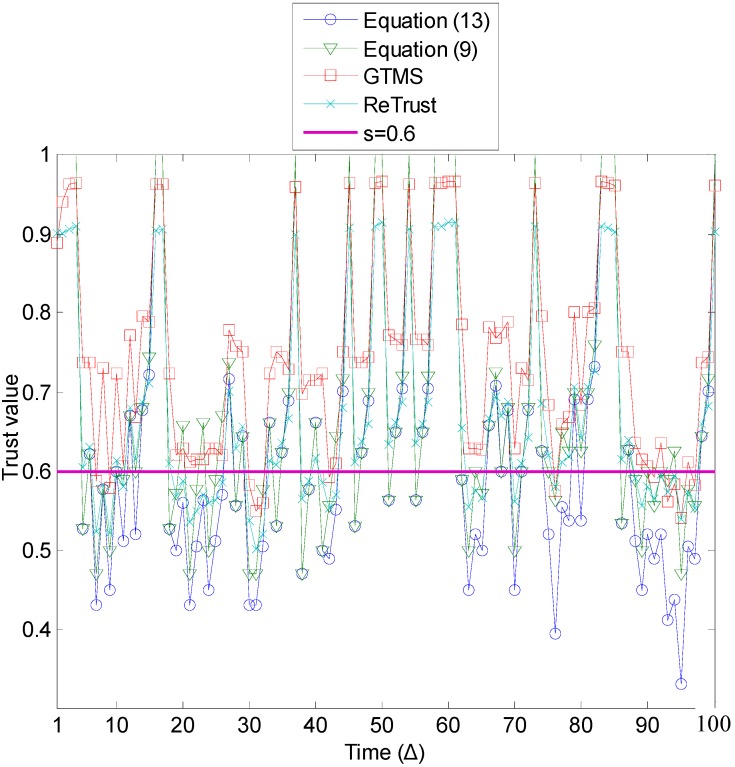
On-off attack detection (probability of an on period is 0.4).

**Figure 11:**
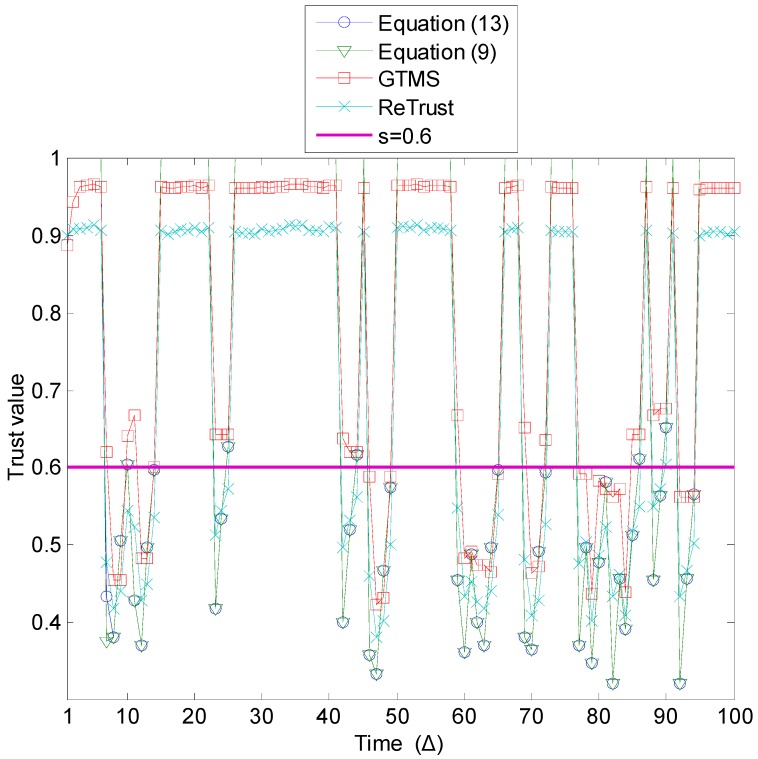
On-off attack detection (probability of an on period is 0.2).

Figure 9, Figure 10 and Figure 11, respectively, show results of the above-defined first, second, and third types of on-off attack.

**Figure 12:**
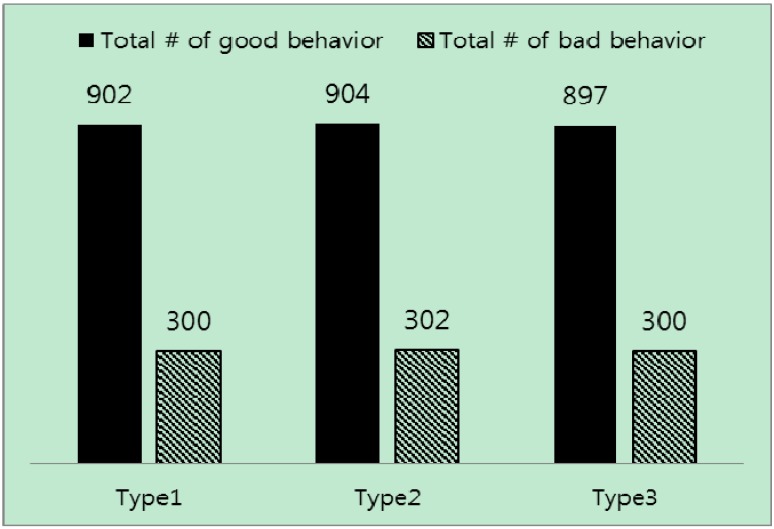
Total incidents of good and bad behavior in three types of on-off attack.

**Figure 13:**
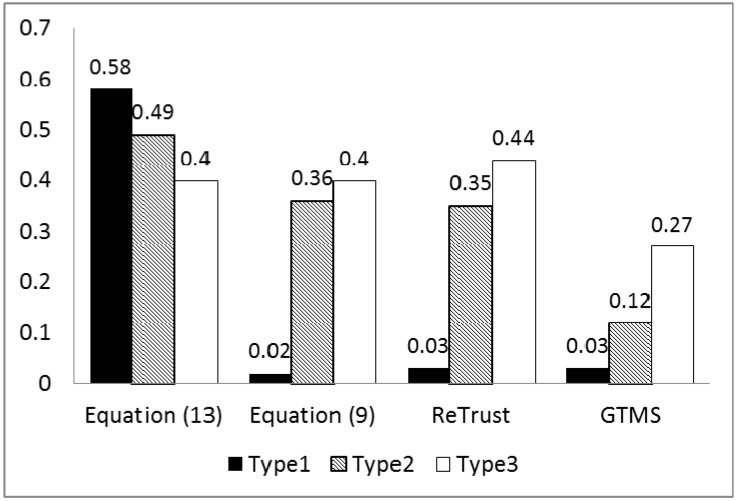
On-off attack detection rate.

An important observation from these three types of evaluation is that, as Figure 12 shows, even though the numbers of good and bad behavior are almost the same equal in all three evaluations, detection rates differ a lot in all trust mechanisms, except in the proposed trust mechanism with Equation (13). For example, in the first type of on-off attack, the detection rate of the trust mechanisms is very low compared to the other types. On the other hand, the detection rate in the proposed trust mechanism with Equation (13) is higher in two types of attack and is stable. Specifically, detection rates of other mechanisms in the first type of attack prove it is necessary to include the misbehavior-frequency component in trust estimation. Moreover, the misbehavior-frequency component also improves attack detection in the second type of attack. As Figure 13 illustrates, the proposed mechanism with Equation (13) outperforms all remaining trust mechanisms in the second type of attack.

## 5. Conclusions

In this paper, we propose a robust and lightweight trust mechanism for WSNs. First, we propose a lightweight trust mechanism that uses only the weight of misbehavior, which is based on a traditional trust estimation design. Then, we enhance our proposed trust mechanism by incorporating a misbehavior-frequency component in order to improve the resiliency of the trust mechanism. Results of the performance evaluation show that the proposed trust mechanism outperforms other trust mechanisms in many aspects. Specifically, general misbehavior detection is higher with the proposed trust mechanism, compared to other trust mechanisms. Moreover, most of the time, the proposed trust mechanism demonstrates a higher and more stable attack detection rate in on-off attacks. But evaluation results show one drawback of the proposed scheme is that it is a bit sensitive to false-positive alarms, compared to other trust mechanisms.

## Appendix

**Table A1:** Definitions of notations.

Notation	Definition
*t_k_*	Time window *k*
*L*	Number of units in the time window
*S_x,y_*	Number of good behavior in node *y* found by node *x*
*U_x,y_*	Number of bad behavior in node *y* found by node *x*
*β*	Weight parameter given to frequency and weight of misbehavior
$T_{t_{k}}$	Trust value in time window $t_{k}$
*α*	Forget factor
$f_{t_{k}}^{m}$	Weight of misbehavior for time window $t_{k}$
$w_{t_{k}}^{m}$	Frequency of misbehavior for time window $t_{k}$
*r_j_*	Rate of misbehavior for time unit *j*
Δ	Duration of one time unit
s	Trust threshold
θ	Threshold for rate of misbehavior

**Table A2:** Computational overhead in terms of number of operations in each trust estimation period.

	Addition	Subtraction	Multiplication	Division	Max	Total
GTMS	2j	0	3	1	0	2j+5
ReTrust	6j−5	2j	2j+1	j+1	0	11j−3
Proposed	j	1	j	j	1	3j+2
Proposed *****	$\left\{ \begin{array}{l} 2j-1\;if\;w_{t_{k}}^{m}>f_{t_{k}}^{m}\\ 2j\;otherwise \end{array}\right.$	$\left\{ \begin{array}{l} 1\;if\;w_{t_{k}}^{m}>f_{t_{k}}^{m}\\ 3\;otherwise \end{array}\right.$	$\left\{ \begin{array}{l} j\;if\;w_{t_{k}}^{m}>f_{t_{k}}^{m}\\ j+2\;otherwise \end{array}\right.$	j+1	1	$\left\{ \begin{array}{l} 4j+2\;if\;w_{t_{k}}^{m}>f_{t_{k}}^{m}\\ 4j+7\;otherwise \end{array}\right.$

*j* is the number of units in the time window. “*Proposed*” is the proposed mechanism without the misbehavior-frequency component. “*Proposed* **^*^**” is the proposed mechanism with the misbehavior-frequency component.
